# Hexavalent Chromium Reduction under Fermentative Conditions with Lactate Stimulated Native Microbial Communities

**DOI:** 10.1371/journal.pone.0083909

**Published:** 2013-12-23

**Authors:** Anil C. Somenahally, Jennifer J. Mosher, Tong Yuan, Mircea Podar, Tommy J. Phelps, Steven D. Brown, Zamin K. Yang, Terry C. Hazen, Adam P. Arkin, Anthony V. Palumbo, Joy D. Van Nostrand, Jizhong Zhou, Dwayne A. Elias

**Affiliations:** 1 ENIGMA – Ecosystems and Networks Integrated with Genes and Molecular Assemblies Program, Lawrence Berkeley National Laboratory, Berkeley, California, United States of America; 2 Biosciences Division, Oak Ridge National Laboratory, Oak Ridge, Tennessee, United States of America; 3 Department of Civil & Environmental Engineering, University of Tennessee, Knoxville, Tennessee, United States of America; 4 Department of Earth & Planetary Sciences, University of Tennessee, Knoxville, Tennessee, United States of America; 5 Department of Microbiology, University of Tennessee, Knoxville, Tennessee, United States of America; 6 Joint Biological Sciences Institute, University of Tennessee, Knoxville, Tennessee, United States of America; 7 Physical Biosciences Division, Lawrence Berkeley National Laboratory, Berkeley, California, United States of America; 8 Institute for Environmental Genomics, Department of Botany, University of Oklahoma, Norman, Oklahoma, United States of America; Instituto Nacional de Cardiologia, Mexico

## Abstract

Microbial reduction of toxic hexavalent chromium (Cr(VI)) *in-situ* is a plausible bioremediation strategy in electron-acceptor limited environments. However, higher [Cr(VI)] may impose stress on syntrophic communities and impact community structure and function. The study objectives were to understand the impacts of Cr(VI) concentrations on community structure and on the Cr(VI)-reduction potential of groundwater communities at Hanford, WA. Steady state continuous flow bioreactors were used to grow native communities enriched with lactate (30 mM) and continuously amended with Cr(VI) at 0.0 (No-Cr), 0.1 (Low-Cr) and 3.0 (High-Cr) mg/L. Microbial growth, metabolites, Cr(VI), 16S rRNA gene sequences and GeoChip based functional gene composition were monitored for 15 weeks. Temporal trends and differences in growth, metabolite profiles, and community composition were observed, largely between Low-Cr and High-Cr bioreactors. In both High-Cr and Low-Cr bioreactors, Cr(VI) levels were below detection from week 1 until week 15. With lactate enrichment, native bacterial diversity substantially decreased as *Pelosinus* spp., and *Sporotalea* spp., became the dominant groups, but did not significantly differ between Cr concentrations. The Archaea diversity also substantially decreased after lactate enrichment from *Methanosaeta* (35%), *Methanosarcina* (17%) and others, to mostly *Methanosarcina* spp. (95%). Methane production was lower in High-Cr reactors suggesting some inhibition of methanogens. Several key functional genes were distinct in Low-Cr bioreactors compared to High-Cr. Among the Cr resistant microbes, *Burkholderia vietnamiensis*, *Comamonas testosterone* and *Ralstonia pickettii* proliferated in Cr amended bioreactors. *In-situ* fermentative conditions facilitated Cr(VI) reduction, and as a result 3.0 mg/L Cr(VI) did not impact the overall bacterial community structure.

## Introduction

Subsurface heavy metal contamination from the nuclear weapons industry is a continuing problem at the Department of Energy site at Hanford, WA. Hexavalent chromium (Cr(VI)) is toxic and highly soluble, and, as a result, can be readily transported through the groundwater [1]. When Cr(VI) is reduced to Cr(III), the solubility and mobility decrease [2], except when Cr(III)-organic complexes can also become soluble and toxic [3]. Microbial Cr(VI)-reduction is one plausible remediation strategy for contaminated sites, with a wide diversity of microorganisms identified to be capable of reducing Cr(VI) as well as other metals [2], [4], [5]. Sustainable long-term microbial Cr(VI)-reduction can be challenging as it is dependent upon several biotic and abiotic processes including physiological, hydrological and geochemical parameters that subsequently control the stability of Cr(III) [6]. Additionally, *in-situ* microbial metal-reduction over time can become inefficient by limited supply of electron donors and acceptors [7], [8], [9].

As an example, polylactate hydrogen release compound (pHRC) was developed and evaluated for overcoming some issues of long term Cr(VI)-reduction for *in-situ* remediation of Cr(VI) contaminated groundwater at the Hanford site [1], [10]. Microcosm experiments stimulated the indigenous microbial community but also shifted the community composition that was able to reduce Cr(VI) [10]. The *in-situ* injection of pHRC into groundwater also stimulated the native microbial community and led to a depletion of higher redox terminal electron acceptors, increasing Fe(II) and significantly decreasing Cr(VI) [1]. Hence, it appeared that in the presence of excess electron donors, the microbial populations may have become more specialized.

Depending upon the type of electron donors and availability of terminal electron acceptors, the stimulated microbial community could be dominated by syntrophs and fermenters, such as *Pelosinus* spp. with lactate enrichment as evidenced in our previous study [11]. *Pelosinus* spp. outcompeted sulfate- and Fe(III)- reducers in a terminal electron limiting environment and some of the species have been shown to reduce variety of metals including Cr(VI) [11], [12], that could be stimulated for metal reduction. Fermentative and methanogenic conditions involve complex syntrophic interactions for stable community structure and function, but not much is known about the impacts of Cr(VI) concentrations on long term stability of fermentative/syntrophic/methanogenic communities and reduction of Cr(VI). It is well established though, that Cr(VI) can be toxic to variety of microorganisms [13], [14]. We anticipated that with exposure to varying levels of Cr(VI), indigenous microorganisms during longer-term lactate enrichment will shift and select for Cr-resistant syntrophic communities. The goals of this study were to determine the lactate enriched native microbial community response to different levels of Cr(VI) concentrations as well as the subsequent Cr(VI)-reduction rate and extent. Continuous flow bioreactors were implemented to enrich native communities from a contaminated site at Hanford, WA with three Cr(VI) concentrations representing the uncontaminated area (0.0 mg/L), the edge of the encroaching Cr plume (0.1 mg/L) and the center of the Cr plume (3.0 mg/L) at the site.

## Materials and Methods

### Native community cultivation and monitoring

Groundwater samples were collected from well 699-96-43 at a depth of 42.5 feet, which is <1 foot from the Hanford formation bedrock, at 100H at the Department of Energy's Hanford Site (Washington, USA) [1]. A description of the site contamination and geochemical history has been previously described [1], [15]. Briefly, Cr(VI) contamination likely emanates from sodium dichromate used for corrosion control at the Hanford plutonium reactors. Cr(VI) groundwater concentrations decrease from the source (area 100D, ∼3.0 mg/L Cr(VI)) to near the shore of the Columbia river (area 100H) at ∼0.1 mg/L Cr(VI). Samples (600 ml) were sealed under N_2_, placed on blue ice packs and shipped to Oak Ridge National Laboratory. Upon arrival, 50 ml was removed as a reference, and immediately frozen at −80°C. The remaining groundwater (550 ml) was inoculated into six custom-built, anaerobic glass fermentation vessels as described previously [11] and in the Supplemental Materials and Methods.

Each vessel received 90 ml of ground water, with working volumes of ∼800 ml with the growth media. Modified CCM media [16] with 30 mM sodium lactate but without exogenous electron acceptors was used for enrichment of the culture (more details are provided in Supplemental Materials and Methods). The duplicate treatments were established where bioreactors 1 and 2 received 30 mM lactate and 0.1 mg/L Cr(VI) (Low-Cr), bioreactors 3 and 4 received 30 mM lactate enrichment only (No-Cr) and bioreactors 5 and 6 received 30 mM lactate and 3.0 mg/L Cr(VI) (High-Cr). The Cr(VI) was added in the form of Na_2_Cr_2_O_7_. The duplicate bioreactors were each supplied with medium from one of three 19 L carboys. Outflow samples from each bioreactor were taken bi-weekly for monitoring growth (OD_600_), pH, Cr(VI), lactate, acetate and butyrate. Growth within the bioreactors was compared to an equal volume taken from the sterile medium carboys. Gas samples were taken aseptically with needles and syringes through vessel top ports sealed with butyl rubber stoppers to measure CO_2_, H_2_ and CH_4_. A separate media outflow sample was taken weekly for Cr(VI) and Fe(III) reduction potential assays. Cr(VI) concentrations were also measured weekly in the samples taken from the “feed carboys” to detect any abiotic reduction of Cr(VI).

### Metabolites, Cr(VI) and metal reduction monitoring in bioreactor samples

Lactate, acetate and butyrate from the media samples were measured using a Waters Breeze 2 HPLC system (Waters Corp., USA). Headspace samples were injected to a Agilent 6850 GC (Agilent Technologies, USA) equipped with a thermal conductivity detector (TCD) for CO_2_ and H_2_ quantification. Samples to detect CH_4_ concentrations were injected into an Agilent 6890 gas chromatograph equipped with a flame ionization detector (FID).

Samples from bioreactors were centrifuged (13,000×g, 8 min, 4°C), cell pellets were washed 3 times with 30 mM lactate/30 mM NaHCO_3_ buffer (pH 6.8) and finally resuspended to 8 ml with the 30 mM lactate/30 mM NaHCO_3_ buffer [17] for Cr(VI) and Fe(III) -reduction assays. The diphenlycarbazide [18] and the ferrozine methods [19], respectively, were used for measuring Cr(VI) and Fe(III) concentrations from the media and resuspended cell pellet samples. Further details on metabolites, and metal-reduction assays are given in the Supplementary Materials and Methods.

### DNA extraction and pyrosequencing of the SSU rRNA subunit of Bacteria and Archaea

For pyrosequencing of the *SSU* rRNA subunit of *Bacteria* and *Archaea*, 13 ml media samples were collected every week from bioreactor outflows, centrifuged and pellets were stored at −80°C until analysis. Total community genomic DNA (cgDNA) was extracted using the PowerSoil™ DNA Isolation Kit (Mo Bio Labs, Inc., Carlsbad, CA). Separate sets of primers targeting the V1-3 hyper variable regions of the SSU rRNA subunit of Bacteria and Archaea were used for PCR amplification of 16S rRNA gene. Primers used for bacteria were 27YMF (AGAGTTTGATYMTGGCTCAG) and 534R (TYACCGCGGCTGCTGG) to get an approximate amplicon length of 431–550 bp, and A2FA (TCYSGTTGATCCYGCSRG) and 571R (GCTACRGVYSCTTTARRC) for Archaea for an approximate amplicon length of 479–1221 [20]. These primers were designed with FLX titanium adapters and 8–10 nt barcodes for sample multiplexing. The PCR reactions were conducted for 10 ng template in 50 µl PCR mix [21] with high fidelity AccuPrime™ *Pfx* DNA polymerase (Invitrogen, Carlsbad, CA). The PCR amplicons were purified using the Agencourt AMPure solid-phase paramagnetic bead technology (Agencourt Bioscience Corporation, Beverly, MA). The PCR amplicon purity, concentration and size were estimated using DNA 1000 reagents and an Agilent 2100 Bioanalyzer (Agilent Technologies, Inc., Waldbronn, Germany). The reactions were paired according to DNA quantity and quality prior to performing emulsion reactions for sequencing on a 454 Life Sciences Genome Sequencer FLX (Roche Diagnostics, Indianapolis, IN) using the unidirection amplicon library sequencing protocol with emPCR Kit II (Roche) and FLX titanium chemistry.

### 16S rRNA amplicon sequence analysis

The 16S rRNA amplicon sequence data was analyzed in MOTHUR version 1.26 [22], QIIME (Quantitative Insights Into Microbial Ecology), version 1.5.0 [23] and AmpliconNoise V1.25 [24]. All samples were run through the AmpliconNoise pipeline to remove sequencing and PCR errors and chimeras using the built-in Perseus algorithm. Sequences were aligned in MOTHUR against the RDP (Ribosomal Database Project) databases for Bacteria and Archaea and trimmed to preserve an approximate average length of 400nt. Pairwise distances were calculated in MOTHUR, which were then clustered based on average linkage clustering. The operational taxonomic units (OTUs) were defined at 97% similarity cutoff for all analyses. BIOM format OTU table was implemented in QIIME pipeline for diversity analysis, classification and assigning taxonomy to the OTUs. Taxonomy reference database from RDP were used for assigning taxonomy to Bacteria and Archaea OTUs. Pairwise Bray-Curtis distances between samples for the OTU table without singletons were used to create non-metric multidimensional scaling plots using the PAST software package [25].

### GeoChip microarray hybridization

Bioreactor DNA samples from selected time points (0.5 w (weeks), 3 w, 6 w, 12 w and 15 w) were submitted to the Institute of Environmental Genomics at Oklahoma University, Norman, OK for GeoChip microarray hybridization. GeoChip 4.0 was used for hybridization. This version contains approximately 152,000 probes covering 410 gene categories [26]. DNA labeling, hybridization, initial data normalization and processing parameters are as previously described [26]. For comparative analysis among the samples, we further normalized the data by log transforming the gene probe signal intensity. NMDS plots for Bray-Curtis dissimilarity distances were calculated in PAST software under default settings. Hierarchical clustering analysis and heat maps were created using heatmap.2 in Gplots package within *R* software [27].

### Sequence accession numbers

Sequences from this study were deposited in the GenBank Short Read Archive database under accession number SRA072678.

## Results

### Microbial community growth with lactate enrichment and Cr(VI) levels

**Figure 1:**
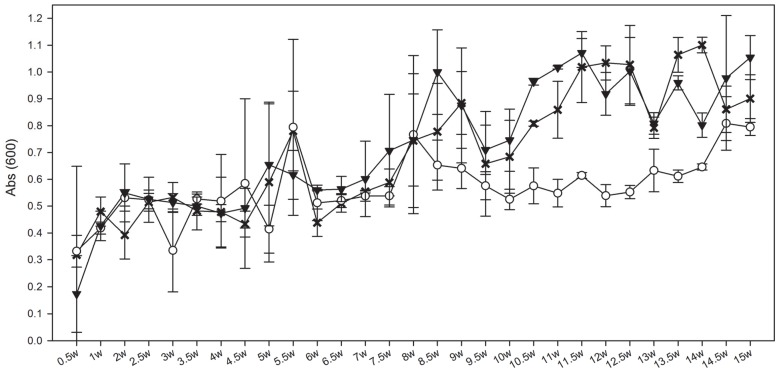
Averaged changes in cell densities (OD_600_) of the microbial consortia in duplicate bioreactors inoculated with Hanford well H-100 groundwater amended with (X) 0 mg/L Cr(VI), (○) 0.1 mg/L Cr(VI) and (▾) 3.0 mg/L Cr(VI). W = weeks. Error bars indicate 1 standard deviation between replicates.

**Figure 2:**
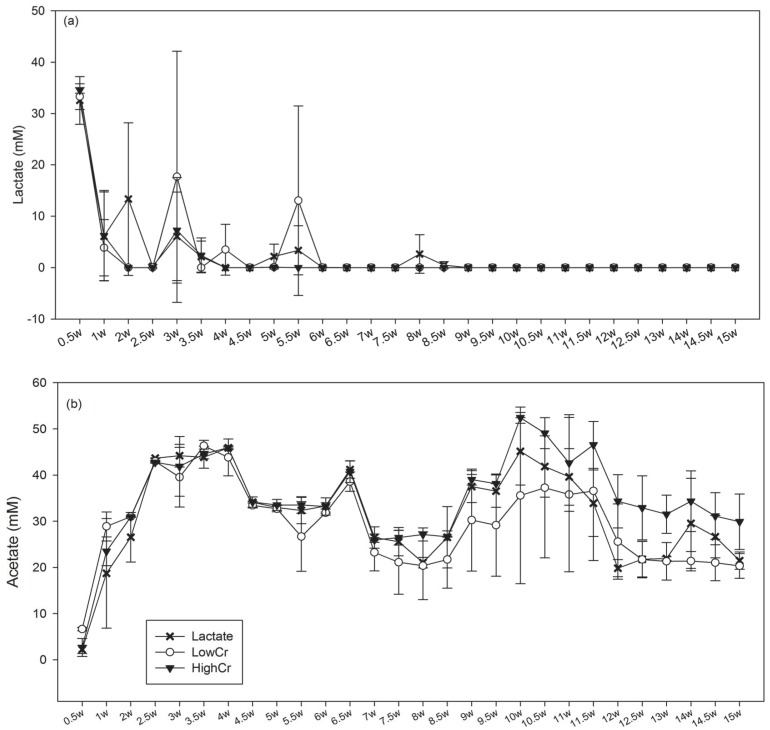
Averaged metabolite concentrations in duplicate bioreactors inoculated with Hanford well H-100 groundwater. (a) Lactate and (b) Acetate concentrations when amended with (X) 0 mg/L Cr(VI), (○) 0.1 mg/L Cr(VI) and (▾) 3.0 mg/L Cr(VI). W = weeks. Error bars indicate 1 standard deviation between replicates.

**Figure 3:**
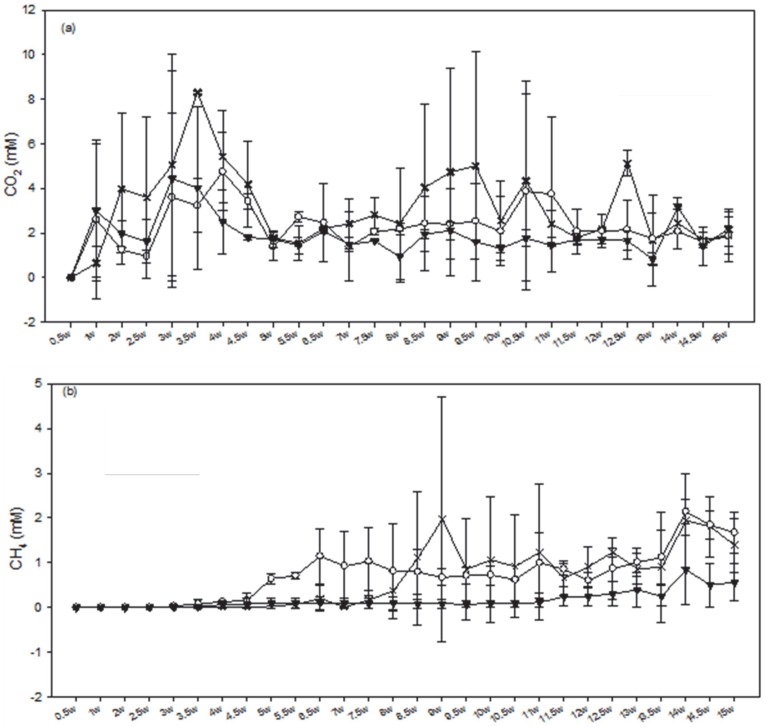
The impact of Cr concentrations on averaged gas concentrations in bioreactors inoculated with Hanford well H-100 groundwater. a) CO_2_ and b) CH_4_ when amended with (X) 0 mg/L Cr(VI), (○) 0.1 mg/L Cr(VI) and (▾) 3.0 mg/L Cr(VI). W = weeks. Error bars indicate 1 standard deviation between replicates.

After 1 week, optical densities (OD_600_) of the communities in all bioreactors were similar (0.39–0.52) and by 7 weeks, all bioreactors had reached densities of 0.56 or greater (Figure 1) and were maintained throughout the experiment. Notable differences were observed in the Low-Cr(VI) (0.1 mg/ml Cr(VI)) bioreactors as they showed greater fluctuation between the replicates early on but maintained a more comparable density during 9–14 weeks. It was notable that these two bioreactors also reproducibly diverged from the others at 8 weeks (0.55–0.65 vs. 0.79–1.1 OD_600_) and it was not until 14.5 weeks that they were statistically (mean standard deviations) indiscernible from the lactate only (0.0 mg/L Cr(VI)) and High-Cr(VI) (3.0 mg/L Cr(VI)) bioreactors (0.77–0.99 OD_600_). Reasons for this are unclear when comparing this disparity in growth from the organic acids (Figure 2) and headspace gas concentrations (Figure 3), as detailed below. Although not statistically significant, the Low-Cr bioreactors did display higher CH_4_ concentrations, however this trend began at 5 weeks (Figure 3B). The pH of all the bioreactors and the media carboys was 7.0+0.2 over the 105 day experiment (data not shown).

Lactate concentrations in the spent media from all bioreactors dropped significantly from ∼35 mM to ∼5 mM after 1.5 weeks and became limited after 4 weeks although fluctuations were observed in individual bioreactors throughout the first 6 weeks (Figure 2A). Acetate concentrations increased to ∼20–30 mM in all bioreactors by week 2, and to 41–46 mM by week 4 (Figure 2B). On week 7, acetate concentrations decreased in all bioreactors to ∼30 mM, but recovered and increased to 40–50 mM. Acetate concentrations in the Low-Cr reactors trended to be lower, although it was not statistically significant. Other metabolites such as butyrate and formate were analyzed, but their presence was intermittent and <2 mM (data not shown).

Headspace CO_2_ concentrations were detected after 1 week between 0.5–3 mM (Figure 3A). Most of the bioreactors maintained 1–4 mM headspace CO_2_ throughout the remainder of the experiment. Some fluctuations were observed throughout the experiment but no consistent trends or significant differences were observed between the Cr levels. By 15 weeks, CO_2_ concentrations in all bioreactors ranged between 1.0–2.7 mM CO_2_. CH_4_ was detected after 3 weeks and maintained a fairly steady concentration ranging between 0.1–1 mM. Substantially higher concentrations were observed in Lactate and Low-Cr bioreactors (0.5–2 mM) compared to High-Cr (0.01–0.15 mM CH_4_).

### Cr(VI)-reduction

**Figure 4:**
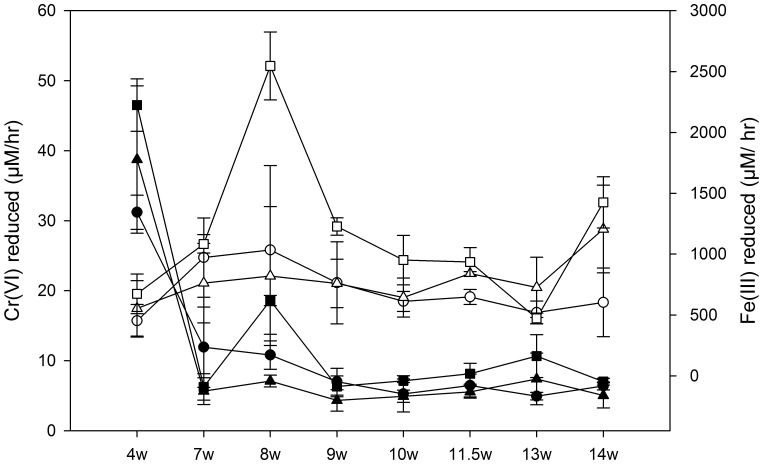
Quantification of metal-reduction capacities of the bioreactor microbial communities over time. Averaged chromium (closed) and soluble iron (open) reduced after twelve hour incubations of washed cells from the duplicate bioreactors inoculated with Hanford well H-100 groundwater amended with (circle) 0.0 mg/L Cr(VI), (triangle) 0.1 mg/L Cr(VI) and (square) 3.0 mg/L Cr(VI). Each time point represents the calculated reduction rate for that weeks sampling from the bioreactors. W = weeks. Error bars indicate 1 standard deviation between replicates.

Immediate reduction of Cr(VI) was observed in Low and High-Cr bioreactors as Cr(VI) was below detection from week 1 through the duration of the experiment (data not shown). Abiotic reduction of Cr(VI) did not occur in the sterile media (data not shown). Washed cells from all bioreactors displayed the ability to reduce Fe(III) and Cr(VI), but in differing amounts throughout the experiment (Figure 4). After 4 weeks, the Cr(VI)-reduction rate in Cr amended bioreactors appeared to be higher than in the lactate only. Washed cells from lactate only bioreactors were able to reduce about 32 µM Cr(VI)/hr, whereas the Low-Cr and High-Cr bioreactor cells were able to reduce about 39 and 47 µM Cr(VI)/h, respectively. However, by 7 weeks, the Cr(VI)-reduction rates significantly dropped to ∼6–8 µM/h in all the bioreactors, and subsequently remained in the range of 4–8 µM/hr. Slightly higher Cr(VI)-reduction rates were observed in High-Cr compared to other bioreactors. Opposite temporal trends were observed for Fe(III)-reduction rates. Between 4 and 8 weeks, the Fe(III)-reduction rates increased from 450 µM/hr to about 1000 µM/hr in lactate only bioreactors, from 550 to 820 µM/hr in Low-Cr and from 675 to 3050 µM/hr in High-Cr bioreactors. After 9 weeks, the Fe(III)-reduction rates decreased in all bioreactors, to 600, 1200 and 2100 µM/hr at 14 weeks in lactate, Low-Cr and High-Cr bioreactors, respectively. Washed cells from High-Cr bioreactors exhibited consistently higher Fe(III) reduction rates than Low-Cr and lactate bioreactors throughout the experiment except at 13 weeks.

### Microbial community composition

**Figure 5:**
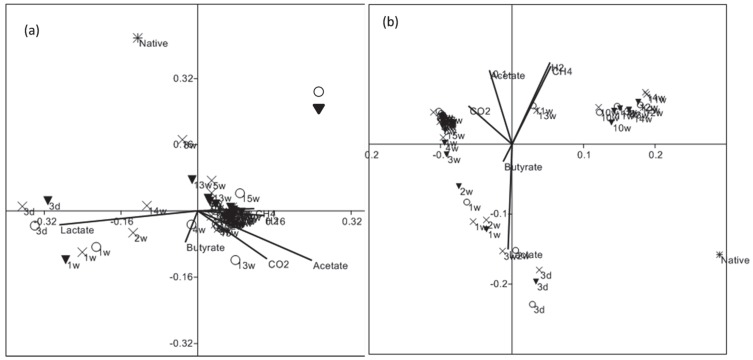
Non-metric multidimensional scaling of OTU composition based on 16S rRNA gene sequences in bioreactors inoculated with Hanford well H-100 groundwater. (a) Bacteria and (b) Archaea amended with (X) 0 mg/L Cr(VI), (○) 0.1 mg/L Cr(VI) and (▾) 3.0 mg/L Cr(VI). W = weeks.

Native microbial community composition from Hanford H-100 ground water significantly changed with lactate enrichment in the continuous flow bioreactors, including the ones amended with Cr(VI). NMDS analysis of Bray-Curtis distances of the rarefied Bacteria and Archaea OTUs between samples showed that major separation occurred between native and lactate amended samples (Figure 5). Microbial communities did not diverge much between Cr amended versus the lactate only controls. Temporal separations were evident initially, as bacterial communities from 3d and 1week time points clustered together. However, after 1week there was not much temporal separation. Similarly the Archaea OTUs at 3d, 1 and 2 weeks did separate from rest of the time points. After two weeks most samples between 10 and 13 weeks clustered together, but no distinct trends were observed between Cr treatments. Lactate, acetate and CO_2_ correlated with bacterial OTUs separation among the metabolites tested. Archaea OTUs separation correlated mostly with lactate, acetate, CH_4_ and H_2_.

**Figure 6:**
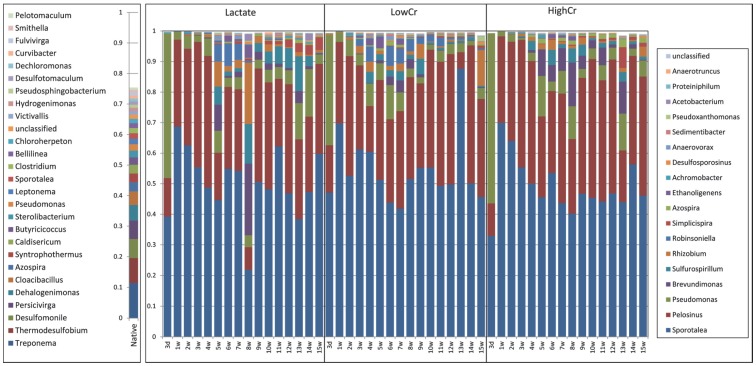
Temporal changes in the relative abundance of the most abundant Bacterial taxa detected by 16S rRNA gene sequencing in bioreactors inoculated with Hanford well H-100 groundwater. Bioreactors were amended with 0/L Cr(VI) (Lactate), 0.1 mg/L Cr(VI) (Low-Cr) and 3.0 mg/L Cr(VI) (High-Cr). W = weeks.

Relative proportions of the predominant bacterial taxa (Figure 6) also showed that native Hanford ground water communities changed significantly with lactate enrichment. Native bacterial communities were more diverse (Shannon diversity = 4.50 (Table S1 in file S1)), with higher relative proportions of *Treponema* (a *Spirochaete*), *Thermodesulfobium* (a *Firmicute*), *Desulfomonile* (a *Deltaprotebacteria*) and *Persivirga* (a *Bacteroidetes*) (Figure 6). *Firmicutes* (18%), *Proteobacteria* (17%) and *Spirochaetes* (13%) were the predominant phyla detected in native environments (Figure S1 in file S1). After 3 days of lactate enrichment, 95% of the communities were *Sporotalea* spp., *Pelosinus* spp. and *Pseudomonas* spp. Among the native communities, they were detected at ∼1.7, 1.4 and 2.1% of the total communities, respectively (Figure S1 in file S1). After 1–4 weeks in the lactate only bioreactor, *Sporotalea* spp. increased to ∼70% of the total community and *Pelosinus* spp. to ∼25–30% of the community. Species diversity within *Veillonellaceae* family was slightly different initially between Cr levels but clustered together after 5 weeks (Figure S2 in file S1). Relative proportions of *Pseudomonas* spp. decreased to <5%.

Similar trends were observed in low and High-Cr bioreactors. After 4 weeks the relative proportions of other bacteria such as *Brevundimonas* spp. *Sulfurospirillum* spp., *Rhizobium* spp., *Robinsoniella* spp. and a few others slightly increased with some temporal variations. These trends were also noticed in Low-Cr bioreactors after 2 weeks and in High-Cr bioreactors after 3 weeks. The diversity indices analysis indicated that the overall diversity of the communities increased at 3–4 weeks. There were no consistently significant differences in communities between Cr amended and the Lactate bioreactors.

**Figure 7:**
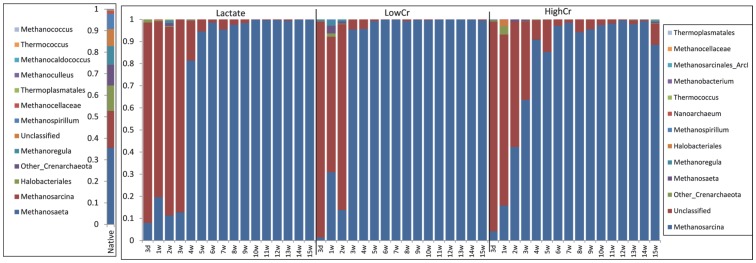
Temporal changes in the relative abundance of the most abundant Archaea detected by 16S rRNA gene sequencing in bioreactors inoculated with Hanford well H-100 groundwater. Bioreactors were amended with 0/L Cr(VI) (Lactate), 0.1 mg/L Cr(VI) (Low-Cr) and 3.0 mg/L Cr(VI) (High-Cr). W = weeks.

The most abundance native Archaea genera in Hanford groundwater sample were *Methanosaeta* (∼35%), *Methanosarcina* (17%), *Halobacteriales* (12%), *Methanoregula* (8%) and others (Figure 7). After lactate enrichment, the Archaeal community composition significantly changed compared to the native community. By 3 days, most of the Archaea were unclassified groups (90%) and the rest were primarily *Methanosarcina*. None of the other predominant Archaea detected in native samples were prevalent after lactate enrichment. By 5 weeks, most of the Archaea classified as *Methanosarcina* (∼95%) and remained until the end of the experiment. *Methanosarcina barkeri* was the closest relative for most of the predominant OTUs observed in bioreactors (Figure S3 in file S1). Similar trends were observed in Cr amended bioreactors. The number of OTUs detected and Shannon diversity index significantly decreased in all reactors compared to native communities (Table S2 in file S1). Archaea diversity continued to decrease over time and was significantly reduced after 5 weeks in lactate only and High-Cr reactors and after 3 weeks in Low-Cr reactors. However, High-Cr reactors established the opposite trend after 10 weeks with significantly higher diversity compared to others.

**Figure 8:**
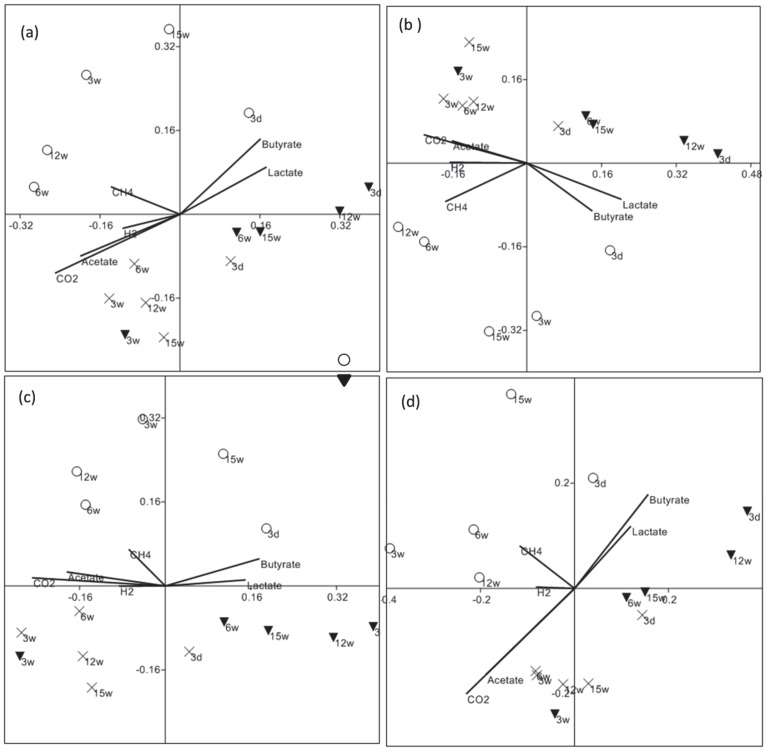
Non-metric multidimensional scaling of Geochip based functional genes composition. (a) all genes detected (b) carbon cycling (c) nitrogen cycling and (d) sulfur cycling genes detected in bioreactors inoculated with Hanford well H-100 groundwater amended with (X) 0 mg/L Cr(VI), (○) 0.1 mg/L Cr(VI) and (▾) 3.0 mg/L Cr(VI). W = weeks.

Microarray hybridization with GeoChip detected several groups of key functional genes including metal resistance, carbon, nitrogen and sulfur cycling genes. NMDS analysis of Bray-Curtis distances of normalized signal intensity of all the functional genes and key groups of genes are presented in Figure 8. Overall the functional gene composition among the bioreactors showed a greater separation of the High-Cr and Low-Cr communities, with the lactate only communities being more similar to High-Cr reactors, suggesting that the low level Cr exposure impacted the community populations moreso than the higher level Cr insult. Separation of the communities between conditions correlated well with CO_2_, acetate and butyrate production and lactate consumption. Similar trends were observed for carbon, nitrogen and sulfur cycling genes, showing that Low-Cr communities were different from High-Cr and Lactate communities. Considerable numbers of chromium resistant bacteria (about 571 positive probes for *chrA* gene (a chromium efflux transporter [28]) were detected in these bioreactors. Hierarchical clustering of the normalized signal intensity of the *chrA* gene probes showed no consistent separation between Cr levels and lactate only bioreactors (Figure S5 in file S1). Some of the predominant Cr resistant bacteria such as *Burkholderia vietnamiensis*, *Comamonas testosteroni*, *Ralstonia pickettii* seem to have proliferated more in Cr amended bioreactors. Other predominant Cr resistant bacteria detected included *Burkholderia phymatum*, *Bradyrhizobium* spp., *Delftia acidovorans*, *Ralstonia solanacearum*, and *Shewanella* spp. Non-metric multidimensional scaling of normalized signal intensity for the *mcrA* gene (the alpha subunit of methyl coenzyme M reductase [29]) detected by GeoChip showed clear separation between Cr levels (Figure S4 in File S1).

## Discussion

The results of this study indicate that a lactate enriched native ground water community from Hanford, WA reduced Cr(VI) and only some Archaea populations were impacted by Cr(VI) concentrations of 0.1 and 3.0 mg/L compared to lactate only treatment. *Pelosinus* spp. and *Sporotalea* spp., both from the family *Veillonellaceae*, were the predominant bacterial groups in all the bioreactors similar to our previous experiment [11]. Cr(VI) concentrations (3.0 and 0.1 mg/L) appeared to have not impacted the growth of these two groups of bacteria, which suggests that some of these species might have evolved Cr resistant strategies [11], [12], and this may be as a result of long term Cr exposure at the Hanford sites. Possibly due to their Cr(VI) resistance, *Pelosinus* spp., have been observed to be one of the predominant groups of microbes detected from several sites with a history of Cr [10], [12], [30] as well as other metal contaminated subsurface environments [31]. It is also interesting that these bacteria were able to outcompete most other primary fermenters, sulfate and iron -reducers which may make these species ideal candidates for bioremediation of Cr impacted subsurface environments, particularly in environments limited by electron-acceptors such as sulfate.

Although overall *Pelosinus* spp. abundance did not diminish throughout the duration of the experiment, the Cr reduction potential of the community significantly decreased in all bioreactors after 4 weeks, which coincided with a slight increase in diversity. Other groups of bacteria such as *Brevundimonas, Sulfurospirillum, Rhizobium, Robinsoniella* were detected at relative proportions of 10–20%, whereas *Veillonellaceae* family OTUs were more similar after 5 weeks. These trends suggest that either specific strains such as *Pelosinus fermentans* A11, that can reduce Cr(VI) (perhaps by respiration) prevailed initially, but later were lost and replaced by species that can only reduce iron, such as A12 and B4 [12].

*Pelosinus* spp. and *Sporotalea* spp. were the predominant primary fermenters of lactate, and possibly to some extent by other groups of bacteria after 4–5 weeks. After 5 weeks, 95% of the Archaea were *Methanosarcina* spp., indicating that these Archaea were one of the predominant groups of organisms able to utilize the by-products of lactate fermentation. It is not clear why only *Methanosarcina* spp. were predominant over others such as *Methanosaeta, Methanoregula, Halobacteriales* and *Methanospirillum*, which were detected at considerable proportions in the native ground water community. One reason could be that members of the *Methanosarcinales*, like *Pelosinus* spp., have a broad substrate spectrum and can grow at sufficient rates due to presence of cytochromes [32] and methanophenazine (a functional menaquinone analogue) [33]. *M. barkeri* was able to efficiently grow on most methanogenic substrates except for formate and was found to have a much higher growth yield on H_2_ and CO_2_ than methanogens without cytochromes [34]. It seems that some cryptic syntrophic relationship may exist between *Pelosinus/Sporotalea* spp. and *M. barkeri*, which should be more fully investigated to understand their ecology and interactions. GeoChip data showed that most of the key biogeochemical cycling gene composition from Low-Cr bioreactors was distinct from the High-Cr and Lactate only bioreactors, which were more similar. This trend suggests that most of the microbial communities represented by GeoChip might be sensitive to lower Cr concentrations. However, it must be noted that only a fraction of the bioreactor microbial community was represented by GeoChip as there were no conserved probes and also a lack of any *Pelosinus* spp. specific probes. Nevertheless, the GeoChip results suggest that Low-Cr concentrations stimulated some stress response compared to the High-Cr concentrations.

The Cr(VI) concentrations were negligible in both Low and High-Cr reactors for the duration of the experiment, even though washed cell assays indicated that Cr(VI)-reduction potential decreased significantly after 4 weeks. These trends open up the possibility that some indirect Cr(VI)-reduction may have occurred via cell metabolites of the fermentative community along with expressed enzymes. A recent study suggested that Cr(VI)-reduction by a *Pelosinus* sp. was catalyzed by indirect mechanisms, as several cellular metabolites such as flavo-proteins were able to reduce Cr(VI) [12], which may have formed some Cr(III)-organic complexes as observed in other studies [35]. Some cellular toxicity to Cr(III) may have also occurred through transient Cr(III) ions [36] or Cr(III)-complexes which can be soluble and toxic [37], thereby possibly explaining the lower methane production observed in high-Cr reactors. Some of the Cr(III) may also be tied up with methanogenic substrates such as acetate, although it appears that other acetotrophic microbes were not impacted as acetate was still metabolized in High-Cr reactors. It seems that the methanogens may be particularly sensitive to Cr(III), an effect that should also be further investigated given the possibly important consequences to bioremediation. It is also critical to evaluate the long term stability of Cr-organic complexes toxicity and reoxidation to Cr(VI), although the latter seems to be unlikely based upon results from this study. Direct enzymatic reduction of Cr(VI) via hydrogenases and cytochromes observed in several microorganisms [38] cannot be completely ruled out as *Pelosinus* spp. contain several hydrogenases and cytochromes [12]. Indirect Cr(VI) reduction by several microbial metabolites is also well documented. For example, under anaerobic conditions, Cr(VI) can be readily reduced to Cr(III) by ferrous iron, sulfides and other low redox metabolites [4], [39]. Perhaps a combination of these factors contributed towards nonspecific Cr(VI) reduction and alleviated toxicity.

## Conclusions

The results of this study showed that lactate enriched native ground water bacterial community from Hanford, WA did not significantly change with exposure to Cr(VI). Stable community structure was maintained with *Pelosinus* spp. and *Sporotalea* spp. and *Methanosarcina* spp. as the predominant groups detected at all three Cr(VI) levels, although CH_4_ production was significantly lower in High-Cr reactors. Cr(VI) was readily reduced in both Low and High-Cr bioreactors, mostly as a result of non-specific reduction by cellular metabolites. Results of this study suggest that low redox conditions with a stable fermentative community stimulated with an electron donor can be a viable option to keep Cr(VI) reduced over the long-term in environments lacking a sufficient supply of terminal electron acceptors.

## Supporting Information

File S1 Contains: Figure S1. Native microbial communities that were detected in Hanford well H-100 groundwater. (a) bacterial phyla and (b) Archaea classes. Figure S2. Non-metric multidimensional scaling of OTUs from *Veillonellaceae* family in bioreactors inoculated with Hanford well H-100 groundwater. Bioreactors were amended with (X) 0 mg/L Na_2_Cr_2_O_7_, (○) 0.1 mg/L Na_2_Cr_2_O_7_ and (▾) 3.0 mg/L Na_2_Cr_2_O_7_. W = weeks. Figure S3. Phylogenetic tree based on neighbor-joining method for predominant Archaea OTUs detected in Hanford well H-100 groundwater sample and in bioreactors inoculated with Hanford well H-100 groundwater. Bioreactors were amended with 0.0 mg/L Na_2_Cr_2_O_7_ (Lactate), 0.1 mg/L Na_2_Cr_2_O_7_ (LowCr) and 3.0 mg/L Na_2_Cr_2_O_7_ (HighCr). Heatmap represents temporal relative abundance. Taxonomic identification was based on NCBI-blast hits. Figure S4. Non-metric multidimensional scaling of normalized signal intensity for *mcrA* gene detected by Geochip in bioreactors inoculated with Hanford well H-100 groundwater.bioreactors were amended with (X) 0 mg/L Na_2_Cr_2_O_7_, (○) 0.1 mg/L Na_2_Cr_2_O_7_ and (▾) 3.0 mg/L Na_2_Cr_2_O_7_. W = weeks. Figure S5. Hierarchical clustering of normalized signal intensity of *chrA* gene probes detected in bioreactors inoculated with Hanford well H-100 groundwater. Bioreactors were amended with (X) 0 mg/L Na_2_Cr_2_O_7_, (○) 0.1 mg/L Na_2_Cr_2_O_7_ and (▾) 3.0 mg/L Na_2_Cr_2_O_7_. W = weeks.
(DOCX)
