# Supplementary material for: Hexavalent Chromium Reduction under Fermentative Conditions with Lactate Stimulated Native Microbial Communities
Source: PLoS One. 2013 Dec 23;8(12):e83909. doi: 10.1371/journal.pone.0083909 (PMC3871698; doi:10.1371/journal.pone.0083909)
Supplement: File S1 — Contains: Figure S1. Native microbial communities that were detected in Hanford well H-100 groundwater. (a) bacterial phyla and (b) Archaea classes. Figure S2. Non-metric multidimensional scaling of OTUs from Veillonellaceae family in bioreactors inoculated with Hanford well H-100 groundwater. Bioreactors were amended with (X) 0 mg/L Na2Cr2O7, (○) 0.1 mg/L Na2Cr2O7 and (▾) 3.0 mg/L Na2Cr2O7. W = weeks. Figure S3. Phylogenetic tree based on neighbor-joining method for predominant Archaea OTUs detected in Hanford well H-100 groundwater sample and in bioreactors inoculated with Hanford well H-100 groundwater. Bioreactors were amended with 0.0 mg/L Na2Cr2O7 (Lactate), 0.1 mg/L Na2Cr2O7 (LowCr) and 3.0 mg/L Na2Cr2O7 (HighCr). Heatmap represents temporal relative abundance. Taxonomic identification was based on NCBI-blast hits. Figure S4. Non-metric multidimensional scaling of normalized signal intensity for mcrA gene detected by Geochip in bioreactors inoculated with Hanford well H-100 groundwater.bioreactors were amended with (X) 0 mg/L Na2Cr2O7, (○) 0.1 mg/L Na2Cr2O7 and (▾) 3.0 mg/L Na2Cr2O7. W = weeks. Figure S5. Hierarchical clustering of normalized signal intensity of chrA gene probes detected in bioreactors inoculated with Hanford well H-100 groundwater. Bioreactors were amended with (X) 0 mg/L Na2Cr2O7, (○) 0.1 mg/L Na2Cr2O7 and (▾) 3.0 mg/L Na2Cr2O7. W = weeks. (DOCX) [file pone.0083909.s001.docx]

**Supplementary information**

**Material and Methods**

***Bioreactor setup***

Six custom-built continuous flow bioreactors were utilized for this study, designed identically to ones used in the previous studies [[1](#_ENREF_1),[2](#_ENREF_2)]. Anaerobic glass fermentation vessels (Allen Glass, Boulder, CO) were used. Each received 90 ml of ground water, with working volumes of ~800 ml. A complete schematic of the experimental design can be found at (<http://aem.asm.org/content/suppl/2012/02/29/78.7.2082.DC1/AEM-AEM07165-11-s01.pdf>). The reactors were supplied with modified CCM medium [[3](#_ENREF_3)] from a single 19-liter carboy (10 liters of medium) via a peristaltic pump at a flow rate of 0.22 to 0.23 ml/min for a dilution rate/medium turnover of 0.487/day. Modified CCM media contained 30 mM sodium DL-lactate, a basal salt solution containing (per liter) 2.17 g NaCl, 5.5 g MgCl_2_.6H_2_O, 0.14 g CaCl_2_ .2H_2_O, 0.5 g NH_4_Cl, and 0.335 g KCl. The medium was buffered using 1.1 mM K_2_HPO_4_ and 30 mM NaHCO_3_ with 1 ml of nonchelated trace elements and 1 ml of a vitamin solution [[4](#_ENREF_4)]. L-Cysteine-HCl (1 mM) and sulfide (1 mM Na_2_S .9H_2_O) were added as reducing agents. Resazurin (1 mg/liter) was added as a redox indicator. Stock solutions of K_2_HPO_4_ (1 M), NaHCO_3_ (6.0 M), L-cysteine .HCl (1 M), Na_2_S .9H_2_O (1 M), and the nonchelated trace element and vitamin mixtures were prepared under anoxic conditions. The medium was made by adding salts, sodium DL-lactate, resazurin to water and then autoclaving. The carboy was kept anaerobic via constant purging with filter-sterilized N_2_ gas. Growth medium was constantly stirred and supplied to the reactors via peristaltic pumps at a flow rate of 0.22-0.23 ml/min for a dilution rate/media turnover of 2.4 d^-1^. The carboys were kept anaerobic via constant purging with filter sterilized N_2_ gas. Anaerobic conditions were maintained with N_2_ gas (7 to 9 ml/min) flushing through the medium inlet drip tube, substantially decreasing biofilm development. Vessel temperature was maintained at 30+2°C by a recirculating water bath. Spent culture fluid and gas drained out of the vessel overflow vents into a closed collection vessel to maintain a constant volume. Exit gas passed through a Zn-acetate solution (1% [wt/vol]) to remove H_2_S before being vented into a chemical fume hood. The pH was maintained at 7.0 to 7.2 by using bicarbonate buffer and small automated additions of 0.1 M NaOH or HCl as needed.

***Microbial growth and metabolite monitoring***

A sample was collected on a bi-weekly basis, filtered and then acidified with 200 mM H_2_SO_4_ (5 mM final concentration) before injection into a Waters Breeze 2 HPLC system (Waters Corp., USA). Metabolites were separated on an Aminex HPX-87H column (BioRad Laboratories) under isocratic temperature (40ºC) and flow (0.5 ml/min), then passed through a refractive index (RI) detector. Metabolite identification used retention time comparison to known standards and quantification was calculated against linear standard curves. All standards were prepared in fresh culture medium to account for any interference of salts in the RI detector.

Fermenter gases were collected via sterilized Hamilton gas-tight syringes and injected into an Agilent 6850 GC (Agilent Technologies, USA) equipped with a thermal conductivity detector (TCD) for CO_2_ quantification. Analytes were separated on an HP-PLOT U column (30m x 0.32 mm x 0.10 μm film, J&W Scientific, Agilent Technologies, USA). Two HP-PLOT U columns were joined together for a total length of 60 m for optimized separation. Samples were injected into a 185ºC split-splitless injector with a split ratio of 3:1 and an isocratic oven (70ºC) with He carrier flow (7.0 ml/min). The detector had 10 ml/min helium makeup flow at 185ºC, with the detector filament set for positive polarity. Samples to detect H_2_ concentrations were injected into a 185°C split-splitless injector with a split ratio of 3:1 and isocratic oven (180°C) and nitrogen carrier flow (3.5 ml/min). The detector had 10 ml/min nitrogen makeup flow at 185°C with the detector filament at negative polarity. Peak identifications were performed by comparison with known standards.

Samples to detect CH_4_ concentrations were injected into an Agilent 6890 gas chromatograph equipped with a flame ionization detector (FID). Samples were separated on a DB-FFAP column (30m x 0.32 mm x 0.5 um film, J&W Scientific, Agilent Technologies, USA) after passing through a 230ºC split-splitless injector with the split ratio set to 3:1 and isocratic oven (50ºC) and helium carrier flow (1.5 ml/min). The FID had a hydrogen flow of 40 ml/min, air at 450 ml/min and helium makeup flow at 45 ml/min. The detector temperature was set at 230ºC. Peak identifications were performed by comparison with known standards and compound quantification was calculated against individual linear standard curves.

***Metal-reduction assays***

Weekly samples (13ml) were taken from the outflow vents of each bioreactor and immediately placed in an anaerobic glove bag unless otherwise noted. Media samples were also taken from the “feed carboys” to ensure reduction had not occurred abiotically. The media samples from bioreactors were centrifuged (13,000 x g, 8 min, 4ºC), cell pellets were washed 3 times with 30 mM lactate/30 mM NaHCO_3_ buffer (pH 6.8) and finally resuspended to 8 ml with the 30 mM lactate/30 mM NaHCO_3_ buffer. Each metal reduction assay (detailed below) was performed in duplicate serum vials (sterile, degassed vials containing 30 mM lactate/30 mM NaHCO_3_ buffer) and contained 2 ml of the resuspended cells. Separate “no cell” and heat-killed controls were employed while *S.* *oneidensis* MR-1 acted as the positive control. Samples were taken at 0, 60, 240 and 720 minutes. Assays for soluble and solid ferric iron-reduction contained 10mM FeCl_3_•6H_2_O and 20 mM FeOOH, respectively and were quantified using the ferrozine method [[5](#_ENREF_5)]. Chromate reduction assays used 60 µM potassium chromate and 60 µM potassium dichromate, individually with the diphenlycarbazide method [[6](#_ENREF_6)]. Average concentrations of Cr(VI) and Fe(III) reduced per hr were calculated for metal-reduction potential analysis. Chromate concentrations were measured in the supernatant media and carboy media using the same method.

Table S1. Temporal changes in observed Bacteria operational taxonomic units (OTUs) and diversity estimates in bioreactors inoculated with Hanford well H-100 groundwater amended with 0 mg/L Na_2_Cr_2_O_7_ (Lactate), 0.1 mg/L Na_2_Cr_2_O_7_ (LowCr) and 3.0 mg/L Na_2_Cr_2_O_7_ (HighCr).

| Diversity  index | Native | 0.5w^¶^ | 1w | 2w | 3w | 4w | 5w | 6w | 7w | 8w | 9w | 10w | 11w | 12w | 13w | 14w | 15w |
| --- | --- | --- | --- | --- | --- | --- | --- | --- | --- | --- | --- | --- | --- | --- | --- | --- | --- |
|  |  | Lactate | | | | | | | | | | | | | | | |
| OTUs | 721 | 41 | 27 | 59 | 49 | 80 | 86 | 114 | 78 | 120 | 78 | 96 | 88 | 95 | 115 | 108 | 266 |
| Shannon | 4.50 | 1.15 | 1.15 | 1.63 | 1.06 | 1.26 | 2.13 | 1.70 | 1.67 | 2.21 | 1.41 | 1.63 | 1.76 | 1.58 | 2.01 | 2.20 | 1.59 |
| Chao1 | 1829 | 110 | 38 | 121 | 85 | 159 | 174 | 261 | 103 | 198 | 120 | 155 | 206 | 171 | 250 | 236 | 791 |
|  |  | LowCr | | | | | | | | | | | | | | | |
|  | OTUs | 68 | 36 | 86 | 88 | 130 | 78 | 77 | 99 | 76 | 55 | 59 | 66 | 81 | 72 | 75 | 395 |
|  | Shannon | 1.25 | 1.28 | 1.25 | 1.41 | 1.96 | 1.52 | 1.90 | 1.93 | 1.55 | 1.50 | 1.46 | 1.55 | 1.47 | 1.51 | 1.28 | 2.06 |
|  | Chao1 | 134 | 74 | 168 | 171 | 343 | 126 | 124 | 162 | 134 | 118 | 87 | 116 | 141 | 127 | 137 | 947 |
|  |  | HighCr | | | | | | | | | | | | | | | |
|  | OTUs | 41 | 34 | 55 | 62 | 78 | 66 | 76 | 81 | 107 | 72 | 76 | 107 | 67 | 114 | 84 | 304 |
|  | Shannon | 1.26 | 1.00 | 1.32 | 1.07 | 1.34 | 1.75 | 1.57 | 1.62 | 1.86 | 1.49 | 1.52 | 1.70 | 1.50 | 2.21 | 1.64 | 1.75 |
|  | Chao1 | 56 | 91 | 109 | 161 | 118 | 110 | 187 | 145 | 218 | 135 | 150 | 205 | 101 | 286 | 135 | 743 |

^¶^w=weeks.

Table S2. Temporal changes in observed Archaea operational taxonomic units (OTUs) and diversity estimates in bioreactors inoculated with Hanford well H-100 groundwater amended with 0 mg/L Na_2_Cr_2_O_7_ (Lactate), 0.1 mg/L Na_2_Cr_2_O_7_ (LowCr) and 3.0 mg/L Na_2_Cr_2_O_7_ (HighCr).

|  | | Native | 0.5w^¶^ | 1w | 2w | 3w | 4w | 5w | 6w | 7w | 8w | 9w | 10w | 11w | 12w | 13w | 14w | 15w |
| --- | --- | --- | --- | --- | --- | --- | --- | --- | --- | --- | --- | --- | --- | --- | --- | --- | --- | --- |
|  |  |  | Lactate | | | | | | | | | | | | | | | |
| OTUs | | 148 | 113 | 130 | 159 | 122 | 115 | 60 | 41 | 55 | 64 | 47 | 13 | 11 | 7 | 22 | 10 | 11 |
| Shannon | | 4.01 | 3.97 | 4.34 | 3.44 | 4.41 | 2.25 | 0.46 | 0.17 | 0.32 | 0.26 | 0.26 | 1.32 | 1.02 | 0.92 | 0.54 | 1.02 | 0.03 |
| Chao1 | | 440 | 169 | 165 | 243 | 196 | 162 | 123 | 53 | 96 | 129 | 142 | 23 | 23 | 9 | 73 | 28 | 14 |
|  | | | LowCr | | | | | | | | | | | | | | | |
|  | OTUs | | 100 | 87 | 139 | 60 | 70 | 20 | 16 | 8 | 35 | 14 | 15 | 6 | 8 | 5 | 7 | 12 |
|  | Shannon | | 1.71 | 3.50 | 4.45 | 0.38 | 0.42 | 0.13 | 0.03 | 0.02 | 0.12 | 0.03 | 1.37 | 0.02 | 0.96 | 1.16 | 0.69 | 0.05 |
|  | Chao1 | | 205 | 125 | 265 | 114 | 183 | 44 | 24 | 12 | 85 | 24 | 29 | 9 | 12 | 7 | 9 | 30 |
|  | | | HighCr | | | | | | | | | | | | | | | |
|  | OTUs | | 77 | 69 | 96 | 166 | 80 | 69 | 35 | 46 | 80 | 30 | 45 | 51 | 19 | 42 | 40 | 51 |
|  | Shannon | | 2.14 | 3.60 | 3.69 | 2.31 | 2.12 | 0.76 | 0.21 | 0.16 | 0.58 | 0.39 | 0.85 | 1.55 | 1.14 | 1.21 | 1.43 | 0.66 |
|  | Chao1 | | 102 | 116 | 153 | 229 | 156 | 95 | 55 | 85 | 208 | 49 | 111 | 82 | 30 | 76 | 111 | 81 |

^¶^w=weeks.

Figure S1.

Figure S2.


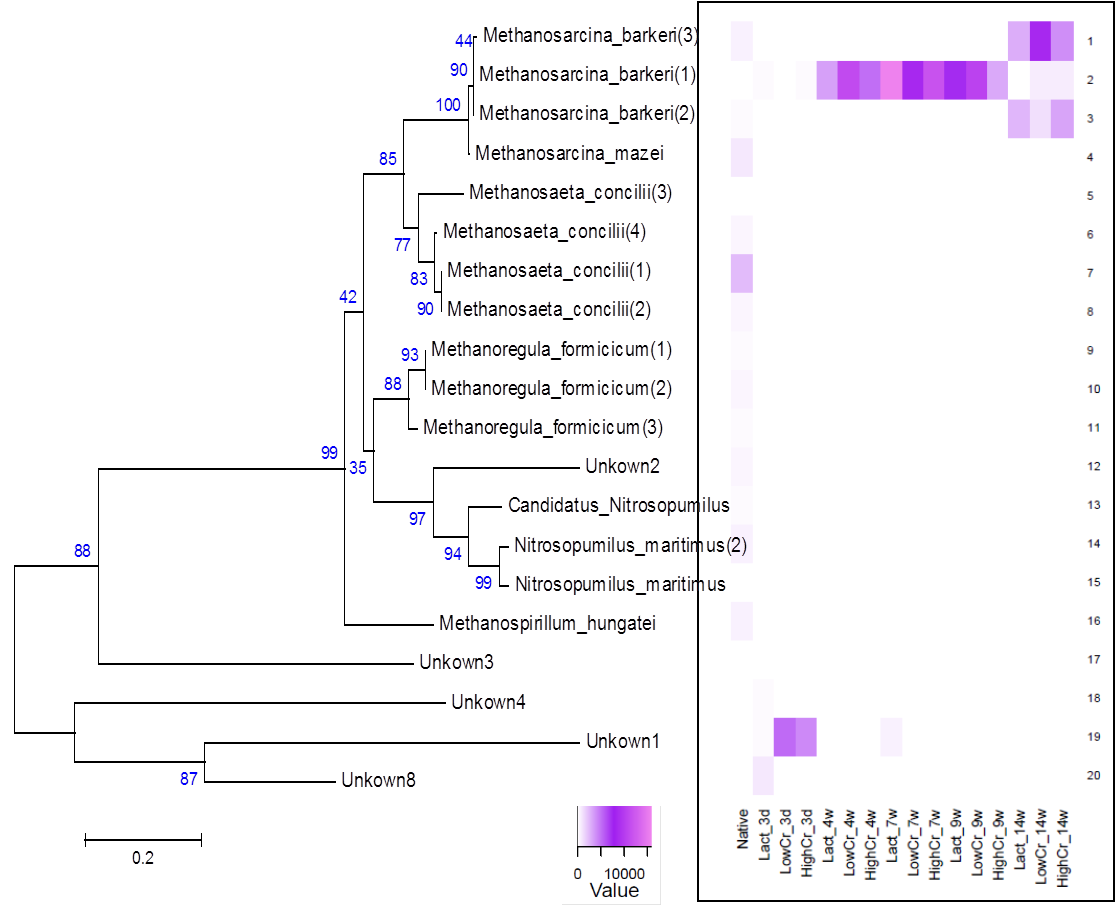
Figure S3.

Figure S4.

Figure S5.

**Supplemental References**

1. Miller LD, Mosher JJ, Venkateswaran A, Yang ZK, Palumbo AV, et al. (2010) Establishment and metabolic analysis of a model microbial community for understanding trophic and electron accepting interactions of subsurface anaerobic environments. BMC Microbiology 10:149.

2. Mosher JJ, Phelps TJ, Podar M, Hurt RA, Campbell JH, et al. (2012) Microbial Community Succession during Lactate Amendment and Electron Acceptor Limitation Reveals a Predominance of Metal-Reducing Pelosinus spp. Applied and Environmental Microbiology 78: 2082-2091.

3. Walker CB, He ZL, Yang ZK, Ringbauer JA, He Q, et al. (2009) The Electron Transfer System of Syntrophically Grown Desulfovibrio vulgaris. Journal of Bacteriology 191: 5793-5801.

4. Brandis A, Thauer RK (1981) Growth of Desulfovibrio Species on Hydrogen and Sulfate as Sole Energy-Source. Journal of General Microbiology 126: 249-252.

5. Lovley DR, Phillips EJP (1987) Rapid assay for microbially reducible ferric iron in aquatic sediments. Applied and Environmental Microbiology 53: 1536-1540.

6. Zhang CL, Liu S, Logan J, Mazumder R, Phelps TJ (1996) Enhancement of Fe(III), Co(III), and Cr(VI) reduction at elevated temperatures and by a thermophilic bacterium. Applied Biochemistry and Biotechnology 57-8: 923-932.
